# Ellagic Acid Alleviates Rheumatoid Arthritis in Rats through Inhibiting MTA1/HDAC1-Mediated Nur77 Deacetylation

**DOI:** 10.1155/2021/6359652

**Published:** 2021-12-09

**Authors:** Huanjin Song, Hao Wu, Jun Dong, Sihua Huang, Jintao Ye, Ruoxi Liu

**Affiliations:** Department of Orthopaedics, The Second Affiliated Hospital of Xi'an Jiaotong University, Xi'an 710004, China

## Abstract

Ellagic acid (EA) was reported to play protective roles in rheumatoid arthritis (RA). It was found that the level of metastasis-associated gene 1 (MTA1)/histone deacetylase 1 (HDAC1) protein complex was downregulated by polyphenols in several human disorders. Notably, inhibition of MTA1 or HDAC1 has anti-inflammatory effects on RA. Therefore, our study is aimed at investigating whether EA prevents RA progression through regulating the MTA1/HDAC1 complex. Herein, the human fibroblast-like synoviocyte (FLS) cell line MH7A was treated with TNF-*α* to induce an inflammation model in vitro and then incubated with different concentrations of EA. Western blot analysis showed that EA reduced MTA1 expression in a dose-dependent manner in MH7A cells. Then, TNF-*α*-treated MH7A cells were incubated with EA alone or together with MTA1 overexpression plasmid (pcDNA-MTA1), and we found that EA inhibited proliferation, inflammation cytokine levels, and oxidative stress marker protein levels and promoted apoptosis in MH7A cells, while MTA1 overexpression abolished these effects. Moreover, coimmunoprecipitation assay verified the interaction between MTA1 and HDAC1. EA downregulated the MTA1/HDAC1 complex in MH7A cells. MTA1 knockdown inhibited proliferation, inflammation, and oxidative stress and promoted apoptosis in MH7A cells, while HDAC1 overexpression reversed these effects. Moreover, chromatin immunoprecipitation assay indicated that EA inhibited HDAC1-mediated Nur77 deacetylation. Rescue experiments demonstrated that Nur77 knockdown reversed the effects of EA on MH7A cell biological behaviors. Additionally, EA treatment attenuated arthritis index, paw swelling, synovial hyperplasia, and inflammation in collagen-induced arthritis (CIA) rats. In conclusion, EA inhibited proliferation, inflammation, and oxidative stress and promoted apoptosis in MH7A cells and alleviated the severity of RA in CIA rats though downregulating MTA1/HDAC1 complex and promoting HDAC1 deacetylation-mediated Nur77 expression.

## 1. Introduction

Rheumatoid arthritis (RA) is a chronic autoimmune disease characterized by synovial tissue inflammation, synovial hyperplasia, articular cartilage destruction, and joint deformities, which results in loss of articular function and seriously affects people's health and quality of life [1]. Fibroblast-like synoviocytes (FLSs) are the predominant cells comprising the synovial membrane structure [2]. Accumulating evidence has suggested that the activated FLSs exhibit similar properties of tumor-like cells, such as hyperproliferation and insufficient apoptosis, which is considered as an essential factor in the occurrence and development of RA [3]. Meanwhile, FLSs can secrete multiple proinflammatory cytokines such as interleukin- (IL-) 6 and IL-1*β* to mediate inflammatory response, which plays a crucial role in articular cartilage destruction [4]. Thus, inhibiting the aberrant activation of FLSs is believed to exert potential therapeutic effects on RA. Current therapies on RA are not effective and can cause serious side effects for RA patients. Therefore, it is necessary to find new strategies for the treatment of RA.

Current evidence has indicated that polyphenols play protective roles in the progression of RA. For instance, kaempferol inhibits the migration and invasion of FLS by blocking the activation of MAPK pathway and attenuated the severity of RA in mice with CIA [5]. Theaflavin-3,3′-digallate significantly reduced arthritis score and incidence in the CIA mouse model and suppressed the expression of inflammatory cytokines in the synovium, which plays preventive role in RA progression via inhibiting the activation of NF-*κ*B and MAPK signaling pathways [6]. Ellagic acid (EA), a natural polyphenol, has a variety of biological activities including antioxidant, anti-inflammatory, and anticancer properties. More notably, it was reported that EA alleviated adjuvant induced arthritis in mice by downregulation of proinflammatory cytokines and upregulation of anti-inflammatory cytokines [7]. Moreover, EA attenuated the severity of adjuvant-induced arthritis in rats through regulating inflammatory signals, oxidative stress, and angiogenesis [8]. However, the underlying mechanism of EA in impeding RA progression remains to be elucidated.

Metastasis-associated gene 1 (MTA1) is extensively identified as an oncogene, and abnormal MTA1 expression is associated with tumor growth, invasion, and metastasis in multiple cancers [9, 10]. In addition, MTA1 is revealed to regulate inflammatory responses and act as a target and a component of NF-*κ*B signaling, which is postulated to be an upstream modulator of inflammation and immunologic responses [11]. More importantly, a previous study illustrated that the expression level of MTA1 was upregulated in human rheumatoid synovial tissues, and knockdown of MTA1 decreased the 4-hydroxynonenal-induced transcriptional expression levels of proinflammatory cytokine genes including IL-1*β*, TNF-*α*, and IL-6 in human FLS MH7A cells [12]. However, the mechanism of MTA1 in regulating inflammation responses in FLSs has been little explored. It was found that MTA1 and the histone deacetylase 1 (HDAC1) proteins are essential components of the nucleosome remodeling and deacetylation (NuRD) complex that mediates gene transcriptional repression [13]. Interestingly, current available studies reported that the level of MTA1/HDAC1 complex could be regulated by polyphenols in human disorders. For instance, pterostilbene suppressed the growth and invasion of hepatocellular carcinoma through effectively inhibiting the levels of the MTA1/HDAC1/NuRD complex and promoting phosphatase and tensin homolog (PTEN) acetylation [14]. Resveratrol downregulated the MTA1/HDAC unit of the NuRD complex and then promoted PTEN acetylation, thus blocking the PTEN/Akt pathway and inhibiting the progression of prostate cancer [15]. Therefore, we speculated that EA, as a polyphenolic compound, might play a preventive role in RA progression by regulating MTA1/HDAC1 complex.

In the present study, we investigated the roles and mechanism of EA in FLS MH7A cells and CIA rat model. And whether EA played functional roles in RA progression by regulating MTA1/HDAC1 complex and its downstream regulatory targets were further explored. This study revealed a novel regulatory mechanism of EA in preventing RA progression.

## 2. Material and Methods

### 2.1. Cell Culture and Treatment

Human FLS cell line MH7A was purchased from American Type Culture Collection (ATCC, Manassas, VA, USA). All cells were cultured in Dulbecco's Modified Eagle's Medium (DEME, Gibco, Grand Island, NY) containing 10% fetal bovine serum (FBS) (Gibco, Carlsbad, CA), 100 U/mL penicillin, and 100 *μ*g/mL streptomycin (Sigma, St. Louis, MO, USA). MH7A cells were incubated in a humidified atmosphere with 5% CO_2_ at 37°C and passaged at about 80% confluence. To induce an inflammation injury model, MH7A cells were stimulated with tumor necrosis factor-*α* (TNF-*α*) (Sigma-Aldrich; 10 ng/mL) for 24 h. For EA treatment, MH7A cells were incubated with different concentrations (10, 25, 50, and 100 *μ*M) of EA (Bidepharm, Shanghai, China) for 24 h.

### 2.2. Cell Transfection

Overexpression plasmids of MTA1 (pcDNA-MTA1) and HDAC1 (pcDNA-HDAC1), small interfering RNAs targeting MTA1 (si-MTA1) and Nur77 (si-Nur77), and their corresponding negative controls (vector and scramble) were obtained from RiboBio Co., Ltd. (Guangzhou, China). When cell population reached to 70-80% confluence, cell transfection was performed by using the Lipofectamine™ 2000 (Invitrogen, Carlsbad, CA) reagent according to the manufacturer's instructions. After transfection for 48 h, cells were collected for further experiments.

### 2.3. RNA Extraction and RT-qPCR

Total RNA of MH7A cells was extracted with TRIzol (Invitrogen, Carlsbad, CA). A Prime Script RT reagent Kit (Takara, Dalian, China) was used to synthesize cDNA. RT-qPCR was performed with SYBR Premix Ex Taq II (Takara, Dalian, China) on an ABI 7500 Real-Time PCR System (Applied Biosystems, Foster City, CA) under the following conditions: 95°C for 1 min, 35 cycles of 95°C for 20 s, 56°C for 10 s, and 72°C for 15 s. The relative expression of Nur77 was normalized to glyceraldehyde 3-phosphate dehydrogenase (GAPDH) and calculated by the 2^-*ΔΔ*CT^ method. The following primer sequences were used: Nur77 (forward, 5′-AAG ATC CCT GGC TTT GCT GAG CTG-3′ and reverse, 5′-AGG CCA GGA TAC TGT CAA TCC AGT-3′) and GAPDH (forward, 5′-CGG AGT CAA CGG ATT TGG TCG TAT-3′ and reverse, 5′-AGC CTT CTC CAT GGT GGT GAA GAC-3′).

### 2.4. Western Blot Analysis

MH7A cells or synovial tissues were lysed in RIPA lysis buffer (Beyotime, Shanghai, China). The total protein concentration was quantified by using BCA Protein Kit (Beyotime, Shanghai, China). Then, equal amount of protein was separated on 10% SDS-PAGE, and the protein bands were transferred to polyvinylidene difluoride (PVDF) membranes (Millipore, Bedford, MA, USA).The membranes were blocked with 5% nonfat milk for 2 h at room temperature and then incubated overnight at 4°C with the following primary antibodies: rabbit polyclonal anti-MTA1 antibody (1 : 1000, ab71153, Abcam), rabbit polyclonal anti-HDAC1 antibody (1 : 1000, ab19845, Abcam), rabbit polyclonal anti-Nur77 antibody (1 : 1000, ab13851, Abcam), and rabbit polyclonal anti-GAPDH antibody (1 : 2500, ab9485, Abcam). Then, the membranes were incubated with horseradish peroxidase- (HRP-) conjugated goat anti-rabbit IgG (1 : 2000, Abcam, ab6721) for 1 h. GAPDH was used as an endogenous control. The protein bands were visualized with ECL detection reagents and analyzed with the ImageJ software (National Institutes of Health, Bethesda, MD, USA).

### 2.5. CCK-8 Assay

Cell Counting Kit-8 (CCK-8) assay (Sigma-Aldrich, St. Louis, MO, USA) was performed to measure MH7A cell proliferation. In brief, MH7A cells were seeded into 96-well microplates. After incubation for 0, 24, 48, and 72 h, 10 *μ*L of CCK-8 solution was added to each well and incubated with MH7A cells for another 2 h at 37°C in a humidified atmosphere with 5% CO_2_. The absorbance at 450 nm of each well was measured by a Microplate Reader (Bio-Rad, Hercules, CA).

### 2.6. Cell Apoptosis Analysis

MH7A cells were collected and stained with the Annexin V-FITC/PI apoptosis detection kit (BD Bioscience, San Jose, CA, USA). Briefly, MH7A cells were resuspended in binding buffer at a density of 1 × 10^6^ cells/mL. Then, 5 *μ*L Annexin V-FITC and 10 *μ*L PI were added into cell suspension and incubated for 15 min in the dark at room temperature. Cell apoptosis was detected with Flow Cytometer (BD Bioscience, San Jose, CA, USA) according to the manufacturer's instruction.

### 2.7. Enzyme-Linked Immunosorbent Assay (ELISA)

MH7A cell supernatant or synovial tissues were collected, and the levels of inflammatory cytokines including TNF-*α*, IL-6, and IL-1*β* were detected by the commercial ELISA kits (R&D systems, USA) according to the manufacturer's instructions.

### 2.8. Coimmunoprecipitation (Co-IP) Assay

MH7A cells were lysed in RIPA buffer containing protease inhibitor (Beyotime, Shanghai, China). Cell supernatant was collected and incubated with anti-MTA1 antibody or IgG at 4°C overnight. Then, the mixture was incubated with 100 *μ*L of protein A/G agarose beads (Takara Biotechnology, Dalian, China) overnight at 4°C. After incubation, the mixture was centrifuged at 1500 g for 5 min, and the beads were collected and washed with PBS for three times. The beads were boiled with loading buffer for 5 min and then centrifuged at 1500 g for 5 min to separate the proteins from beads. Then, the supernatant was collected, and the obtained proteins were subsequently analyzed by using Western blot analysis.

### 2.9. Chromatin Immunoprecipitation (ChIP)

Chromatin immunoprecipitation (ChIP) analysis was performed with the EZ-Magna ChIP TMA kit (Millipore, Billerica, MA) according to the manufacturer's instructions. Briefly, MH7A cells in the exponential phase of growth were crosslinked with 1% formaldehyde for 10 min, followed by reaction with 125 mmol/L glycine at room temperature for 5 min. Then, cells were centrifuged at 1000 g for 5 min, washed twice with PBS, and suspended in cell lysate at a density of 2 × 10^6^ cells per 200 *μ*L. A mixture of protease inhibitors was added to the cell suspension, and cells were then centrifuged and resuspended with nuclear separation buffer, followed by ultrasonic treatment to obtain 200-1000 bp chromatin fragments. The fragments were centrifuged at 14,000 g at 4°C for 10 min, and the supernatant was collected. 100 *μ*L of supernatant was incubated with 900 *μ*L of ChIP Dilution Buffer, 20 *μ*L of 50 × PIC, and 60 *μ*L of Protein Agarose/Salmon Sperm DNA at 4°C for 1 h and then centrifuged to collect the supernatant. The supernatant was incubated with anti-HDAC1 antibody or IgG at 4°C overnight and then centrifuged at 1000 g for 10 min to collect the pellet. The precipitated DNA fragments in the complex were quantified by using RT-qPCR.

### 2.10. Oxidative Marker Protein Assessment

Oxidative damage is determined by measuring the levels of malondialdehyde (MDA) and superoxide dismutase (SOD). In brief, MH7A cells were exposed to 6.25 *μ*g/mL c-BC and/or 4 *μ*g/mL for 24 h, washed once with ice-cold PBS, and then lysed in RIPA lysis buffer (Beyotime, Shanghai, China) for 30 min. The lysates were centrifuged, and the supernatant was collected for the assessment of the levels of SOD and MOD. The protein concentration of the supernatant was examined by using the BCA Protein Kit (Beyotime, Shanghai, China). All examinations were performed by using commercial MDA and SOD assay kits (Jiancheng Bioengineering institute, Nanjing, China) according to the manufacturer's instructions.

### 2.11. Animal Experiments

Healthy male Wistar rats (160-170 g) aged six-week-old were obtained from the Experimental Animal Center of the Second Affiliated Hospital of Xi'an Jiaotong University (Xi'an, China), which were remained in sterile cages under standard laboratory conditions (12 h light/dark cycle, 22-25°C temperature, 55-60% humidity) with unrestricted food and water. The rats were randomly assigned to five groups (*n* = 8 per group): control, CIA, CIA + EA (25 mg/kg), CIA + EA (50 mg/kg), and CIA + EA (100 mg/kg). To establish a CIA model, bovine collagen type II (CII) was dissolved in 0.05 mol/L acetic acid to a final concentration of 2 mg/mL and vortexed overnight at 4°C. Subsequently, this solution was emulsified on ice with an equal volume of complete Freund's adjuvant (Sigma-Aldrich, MO, USA) to form a stable emulsion of 1 mg/mL. Rats were immunized by intradermally injecting 200 *μ*L of the collagen emulsion at their back base of tails on day 0, followed by booster injection of the same dose of the emulsion on day 7. And equal volumes of sterilized saline were injected in rats of the control group. For EA treatment groups, CIA rats were orally administered with EA (25, 50, and 100 mg/kg) once a day from day 21. Concomitantly, the rats in the control and CIA groups were orally administered with the same volume of sterilized saline using the same method. All rats were euthanized on day 42, and the synovial joint tissues were collected for subsequent experiments. All experiments were conducted in accordance with the international guidelines of the Animal Care and Use Committee and approved by the Animal Ethics Committee of the Second Affiliated Hospital of Xi'an Jiaotong University.

The arthritic index was used to evaluate the severity of arthritis, which was scored once per week from the first immunization. A scoring system was used to assess the severity of CIA. Scoring was as follows: 0, no swelling or redness; 1, slight swelling and redness in the tarsals or ankle joints; 2, mild range of swelling and redness from the ankle to the tarsals; 3, serious swelling and redness extending from the ankle to metatarsal joints; and 4, the most severe degree of swelling and redness encompassing the ankle, foot, and digits or ankylosis of the limb. The arthritic index was defined as the sum of the scores of four paws of each rat and recorded as the mean score obtained by three experimental operators. An arthritis index over 6 points can be judged to indicate the successful establishment of the CIA model. The swelling degree of the paws was estimated by using a plethysmometer to measure paw volume every week.

### 2.12. Histopathological Analysis

The synovial joint tissues were collected and fixed in 10% neutral buffered formalin and then embedded in paraffin and sliced. Paraffin sections were stained with hematoxylin and eosin (H&E) or safranin-fast green staining using a standard protocol and analyzed by light microscopy.

### 2.13. Statistical Analysis

Statistical analysis was performed with the SPSS version 22.0 software. All data from at least three replicate experiments were presented as mean ± standard deviation (SD). Student's *t*-test and analysis of variance (ANOVA) were performed for the comparison between two groups and comparison among groups, respectively. *P* < 0.05 was considered as statistically significant.

## 3. Results

### 3.1. EA Reduced MTA1 Expression Level in MH7A Cells

**Figure 1:**
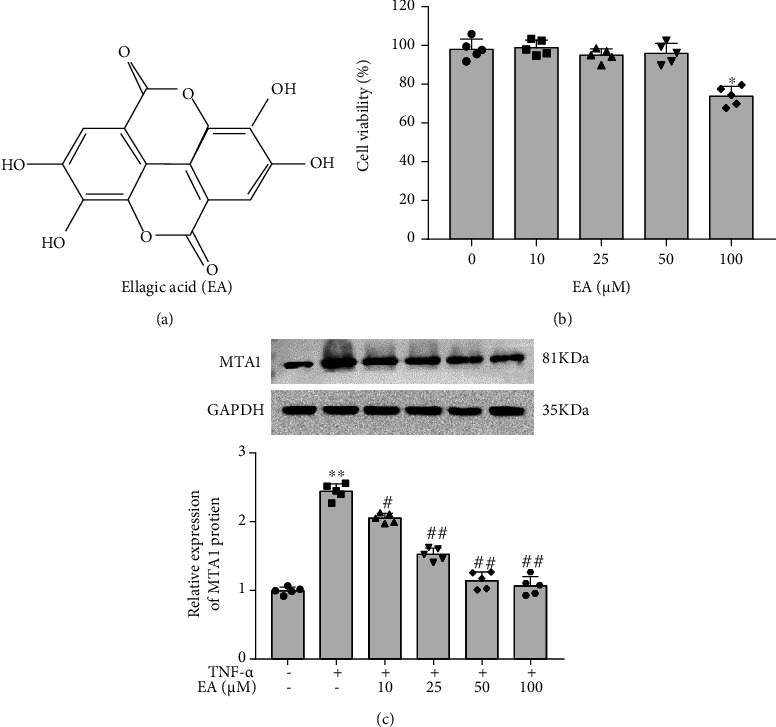
EA reduced MTA1 expression in MH7A cells. (a) The molecular formula of EA. (b) MH7A cells were incubated with different concentrations (0, 10, 25, 50, and 100 *μ*M) of EA. MH7A cell viability was measured by using CCK-8 assay. ^∗^*P* < 0.05 compared with 0 *μ*M EA group. (c) MH7A cells were treated with 10 ng/mL TNF-*α* to induce inflammation injury and then incubated with different concentrations of EA. Western blot analysis was performed to evaluate the expression of MTA1 protein. Data from at least three replicate experiments were presented as mean ± SD. *N* = 5. ^∗∗^*P* < 0.01 compared with the first group, ^#^*P* < 0.05, ^##^*P* < 0.01 compared with the second group.

To explore the role of EA in regulating MH7A cell functions, MH7A cells were treated with different concentrations (0, 10, 25, 50, and 100 *μ*M) of EA. The molecular formula of EA is shown in Figure 1(a). CCK-8 assay indicated that 10, 25, and 50 *μ*M EA have no effect on MH7A cell viability, but 100 *μ*M EA impaired cell viability (Figure 1(b)). Thus, 50 *μ*M was selected as the EA treatment condition in subsequent experiments. MH7A cells were treated with TNF-*α* to induce inflammation model and then incubated with different concentrations of EA. Western blot analysis showed that MTA1 expression level was increased obviously after TNF-*α* treatment, while EA incubation reduced MTA1 expression level in a dose-dependent manner in MH7A cells (Figure 1(c)).

### 3.2. EA Inhibited Proliferation, Inflammation, and Oxidative Stress and Promoted Apoptosis by Downregulating MTA1 in MH7A Cells

**Figure 2:**
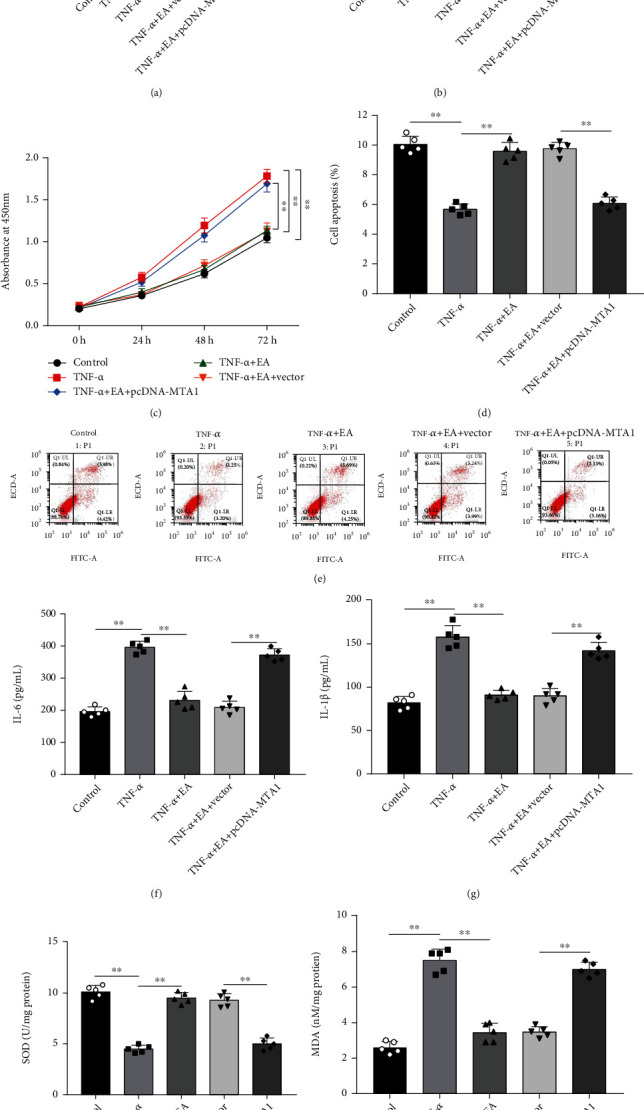
EA inhibited proliferation, inflammation, and oxidative stress and promoted apoptosis by downregulating MTA1 in MH7A cells. TNF-*α*-treated MH7A cells were treated with 50 *μ*M EA alone or together with pcDNA-MTA1. (a, b) The expression of MTA1 protein was detected by Western blot analysis. (c) CCK-8 assay was performed to measure MH7A cell proliferation. (d, e) MH7A cell apoptosis was measured by flow cytometry. (f, g) The levels of IL-6 and IL-1*β* in cell culture supernatant were detected by ELISA. (h, i) The levels of SOD and MDA in MH7A cells were examined by commercial kits. Data from at least three replicate experiments were presented as mean ± SD. *N* = 5. ^∗∗^*P* < 0.01.

To investigate whether EA affects MH7A cells through regulating MTA1 expression, TNF-*α*-treated MH7A cells were incubated with 50 *μ*M EA alone or together with pcDNA-MTA1. Western blot analysis showed that EA reduced MTA1 expression in TNF-*α*-treated MH7A cells, while transfection of pcDNA-MTA1 increased MTA1 expression (Figures 2(a) and 2(b)). Moreover, EA treatment inhibited proliferation (Figure 2(c)) and promoted apoptosis (Figures 2(d) and 2(e)) in TNF-*α*-treated MH7A cells, while MTA1 overexpression reversed these effects. Furthermore, TNF-*α* treatment increased the levels of inflammatory cytokines IL-6 and IL-1*β* in MH7A cells, and EA inhibited the levels of IL-6 and IL-1*β*, while MTA1 overexpression abolished these effects (Figures 2(f) and 2(g)). Besides, we found that TNF-*α* treatment decreased SOD level (Figure 2(h)) and increased MDA level in MH7A cells (Figure 2(i)), while EA treatment enhanced SOD level and reduced MDA level, which were then reversed by MTA1 overexpression. These results indicated that EA inhibited proliferation, inflammation, and oxidative stress and promoted apoptosis by downregulating MTA1 in MH7A cells.

### 3.3. MTA1 Interacted with HDAC1 and Promoted HDAC1 Expression in MH7A Cells

**Figure 3:**
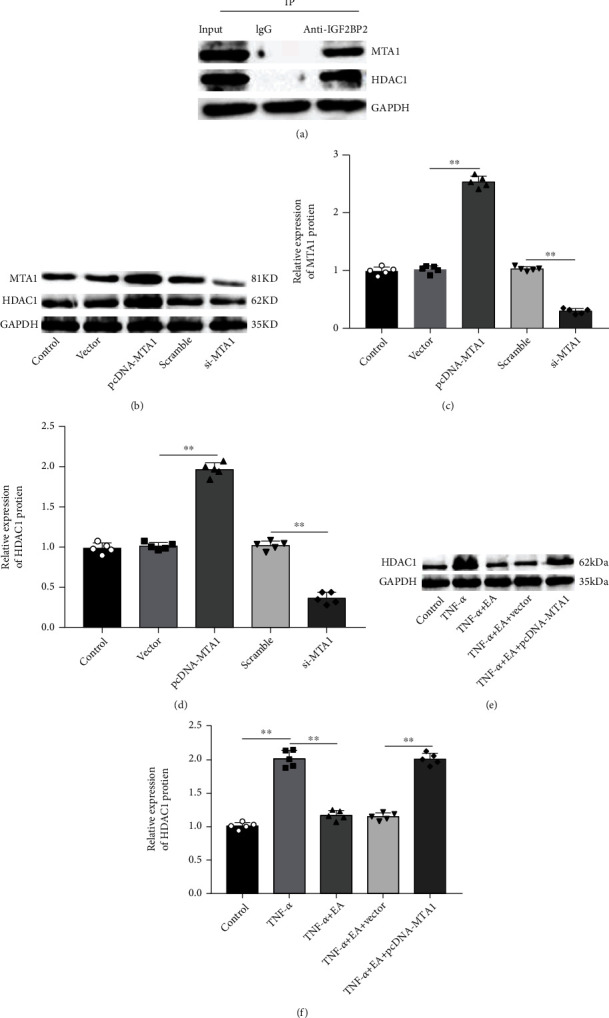
MTA1 interacted with HDAC1 and promoted HDAC1 expression. (a) The interaction between MTA1 protein and HDAC1 protein was verified by Co-IP assay. (b–d) pcDNA-MTA1, si-MTA1, and their negative controls were transfected into MH7A cells, respectively. The expression of MTA1 and HDAC1 was measured by Western blot analysis. (e, f) TNF-*α*-treated MH7A cells were treated with 50 *μ*M EA alone or together with pcDNA-MTA1. The expression of HDAC1 was measured by Western blot analysis. Data from at least three replicate experiments were presented as mean ± SD. *N* = 5. ^∗∗^*P* < 0.01.

A previous study has validated that MTA1 and HDAC1 proteins are essential components of the NuRD complex [13]. Herein, Co-IP assay was performed to verify the interaction between MTA1 and HDAC1 proteins in MH7A cells, which indicated that both MTA1 and HDAC1 proteins could be detected by immunoprecipitation with MTA1 antibody but not with IgG in MH7A cells (Figure 3(a)). Moreover, MH7A cells were transfected with pcDNA-MTA1 or si-MTA1, and the overexpression and interfering efficiencies were determined by Western blot analysis (Figures 3(b) and 3(c)). And we observed that MTA1 overexpression significantly increased HDAC1 expression, while MTA1 knockdown decreased HDAC1 expression in MH7A cells (Figures 3(b) and 3(d)). Besides, we further found that TNF-*α* treatment induced HDAC1 expression in MH7A cells, and EA reduced HDAC1 expression, while transfection of pcDNA-MTA1 increased HDAC1 expression (Figures 3(e) and 3(f)).

### 3.4. MTA1 Knockdown Inhibited Proliferation, Inflammation, and Oxidative Stress and Promoted Apoptosis by Downregulating HDAC1 in MH7A Cells

**Figure 4:**
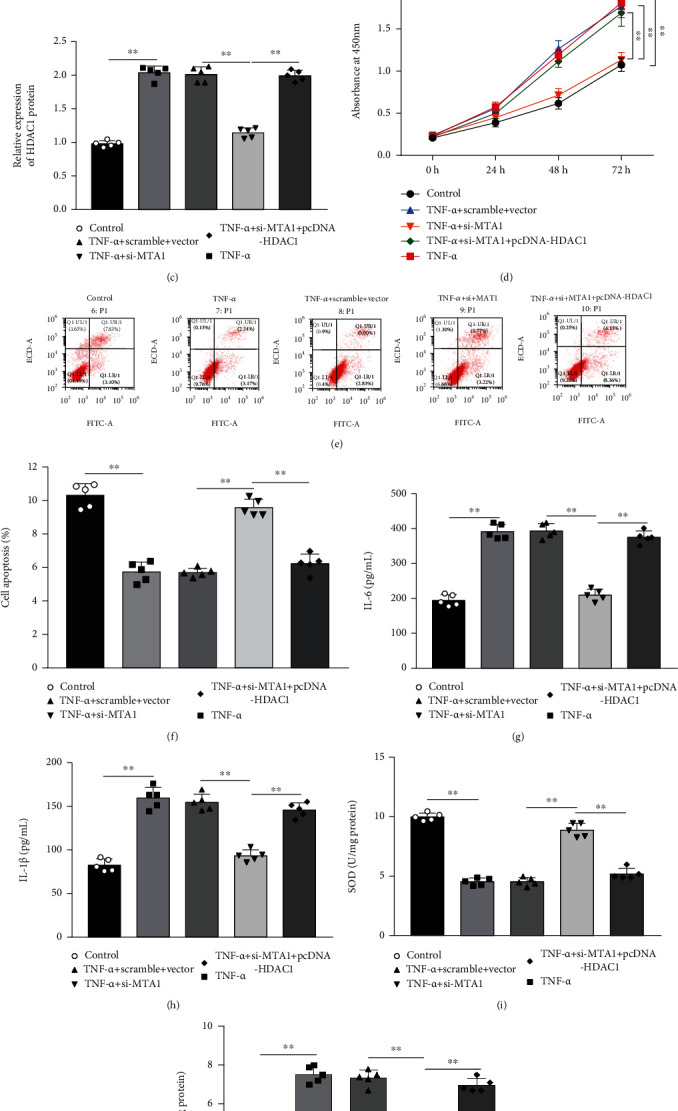
MTA1 knockdown inhibited proliferation, inflammation, and oxidative stress and promoted apoptosis by downregulating HDAC1 in MH7A cells. TNF-*α*-treated MH7A cells were transfected with si-MTA1 alone or together with pcDNA-HDAC1. (a–c) The protein expression of MTA1 and HDAC1 was detected by Western blot analysis. (d) MH7A cell proliferation was measured by CCK-8 assay. (e, f) Flow cytometry was performed to measure MH7A cell apoptosis. (g, h) The levels of IL-6 and IL-1*β* in cell culture supernatant were detected by ELISA. (i, j) The levels of SOD and MDA in MH7A cells were examined by commercial kits. Data from at least three replicate experiments were presented as mean ± SD. *N* = 5. ^∗∗^*P* < 0.01.

To explore the roles of MTA1/HDAC1 in regulating MH7A cell functions, TNF-*α*-treated MH7A cells were transfected with si-MTA1 alone or together with pcDNA-HDAC1. Western blot analysis showed that transfection of si-MTA1 decreased the expression levels of MTA1 and HDAC1 proteins in TNF-*α*-treated MH7A cells, while transfection of pcDNA-HDAC1 increased their expression (Figures 4(a)–4(c)). Moreover, it was observed that MTA1 knockdown suppressed cell proliferation (Figure 4(d)) and promoted apoptosis (Figures 4(e) and 4(f)) in TNF-*α*-treated MH7A cells, which were then reversed by HDAC1 overexpression. Besides, MTA1 knockdown attenuated the levels of IL-6 and IL-1*β* in TNF-*α*-treated MH7A cells, while HDAC1 overexpression reversed these effects (Figures 4(g) and 4(h)). Additionally, we found that MTA1 knockdown enhanced SOD level (Figure 4(i)) and reduced MDA level (Figure 4(j)) in TNF-*α*-treated MH7A cells, which were reversed by HDAC1 overexpression. These results revealed that MTA1 knockdown inhibited proliferation, inflammation, and oxidative stress and promoted apoptosis by downregulating HDAC1 in MH7A cells.

### 3.5. EA Inhibited HDAC1-Mediated Nur77 Deacetylation in MH7A Cells

**Figure 5:**
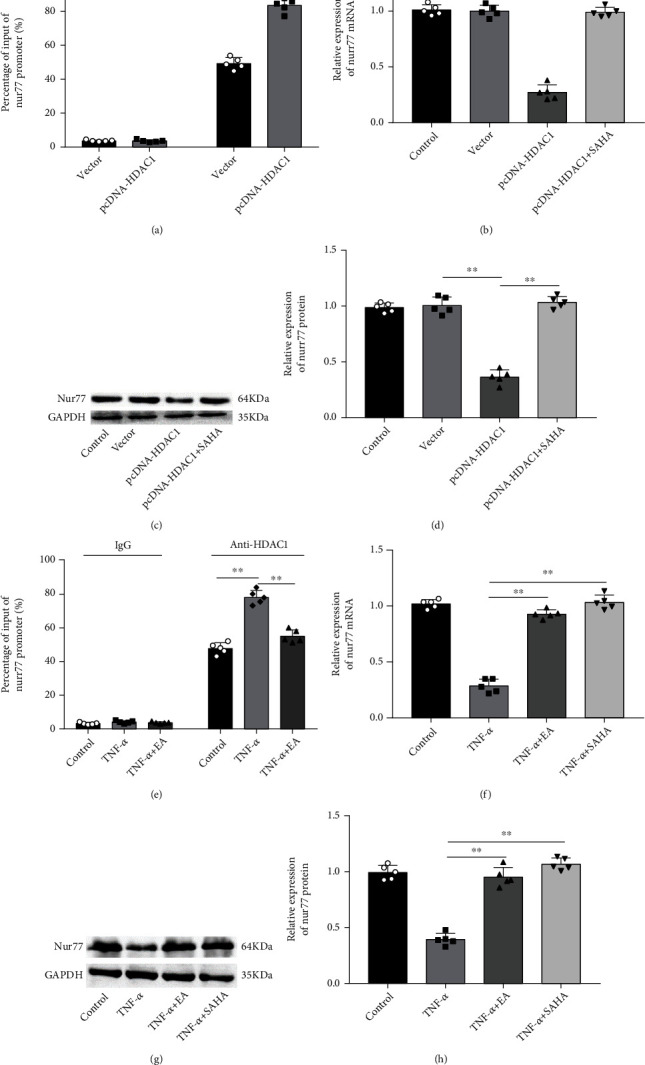
EA inhibited HDAC1-mediated Nur77 deacetylation in MH7A cells. (a) ChIP was employed to detect the enrichment of HDAC1 on the Nur77 promoter region in MH7A cells after HDAC1 overexpression. (b–d) MH7A cells we transfected with pcDNA-HDAC1 alone or together with treatment with 5 *μ*M HDAC1 inhibitor SAHA, the mRNA and protein expression of Nur77 was detected by RT-qPCR and Western blot analysis, respectively. (e) TNF-*α*-treated MH7A cells were treated with 50 *μ*M EA; ChIP was employed to detect the enrichment of HDAC1 in the Nur77 promoter region in MH7A cells. (f–h) The mRNA and protein expression of Nur77 was detected by RT-qPCR and Western blot analysis, respectively. Data from at least three replicate experiments were presented as mean ± SD. *N* = 5. ^∗∗^*P* < 0.01.

An available study verified that Nur77 expression was regulated by HDAC1-mediated deacetylation [16]. ChIP assay was firstly performed to verify this regulatory mechanism in MH7A cells, which showed that HDAC1 was enriched in the promoter region of Nur77, and HDAC1 overexpression promoted the enrichment of HDAC1 in Nur77 promoter (Figure 5(a)). Subsequently, MH7A cells we transfected with pcDNA-HDAC1 alone or together with treatment with 5 *μ*M HDAC1 inhibitor suberoylanilide hydroxamic acid (SAHA), it was observed that transfection of pcDNA-HDAC1 significantly decreased the mRNA and protein expression levels of Nur77, while SAHA abolished these effects in MH7A cells (Figures 5(b)–5(d)). Moreover, ChIP assay displayed that the enrichment of HDAC1 on Nur77 promoter was enhanced in MH7A cells after TNF-*α* treatment, which was then reduced by EA treatment (Figure 5(e)). Additionally, we found that TNF-*α* treatment decreased the mRNA and protein expression of Nur77, while both EA and SAHA treatment increased Nur77 expression (Figures 5(f)–5(h)). Collectively, the obtained results demonstrated that EA inhibited HDAC1-mediated Nur77 deacetylation in MH7A cells.

### 3.6. Nur77 Knockdown Reversed the Effects of EA on Proliferation, Inflammation, Oxidative Stress, and Apoptosis in MH7A Cells

**Figure 6:**
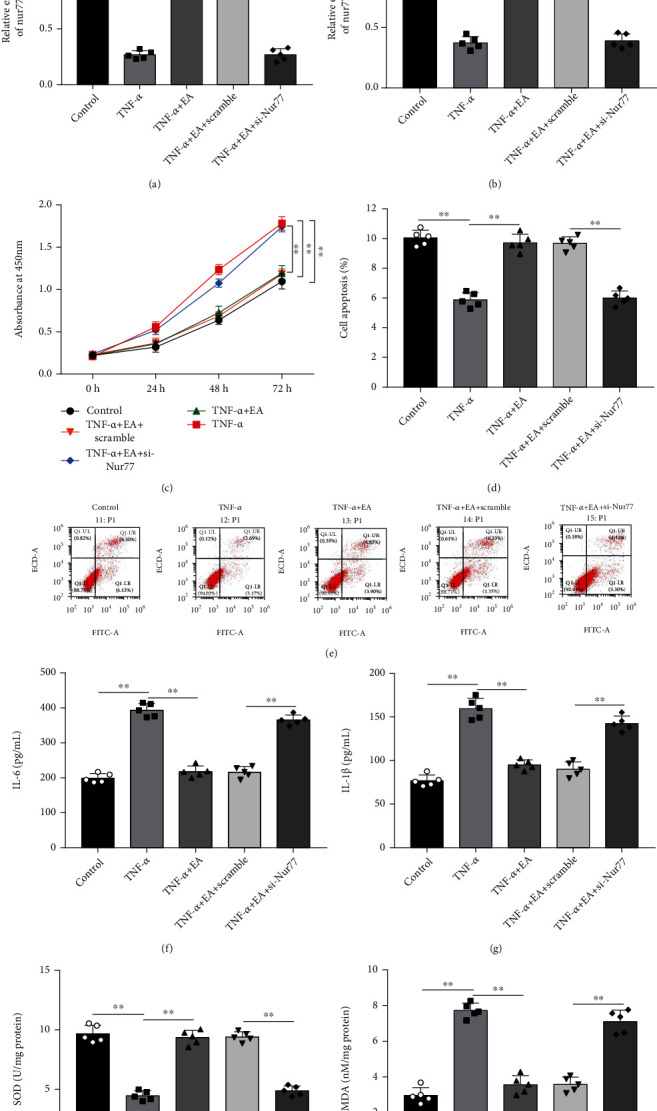
Nur77 knockdown reversed the effects of EA on proliferation, inflammation, oxidative stress, and apoptosis in MH7A cells. TNF-*α*-treated MH7A cells were treated with 50 *μ*M EA alone or together with si-Nur77. (a) The mRNA expression of Nur77 was detected by RT-qPCR. (b) The protein expression of Nur77 was detected by Western blot analysis. (c) CCK-8 assay was performed to measure MH7A cell proliferation. (d, e) MH7A cell apoptosis was measured by flow cytometry. (f, g) The levels of IL-6 and IL-1*β* in cell culture supernatant were detected by ELISA. (h, i) The levels of SOD and MDA in MH7A cells were examined by commercial kits. Data from at least three replicate experiments were presented as mean ± SD. *N* = 5. ^∗∗^*P* < 0.01.

To investigate whether EA plays functional roles in MH7A cells through indirectly regulating Nur77 expression, TNF-*α*-treated MH7A cells were incubated with 50 *μ*M EA alone or together with si-Nur77. We observed that TNF-*α* treatment reduced the mRNA and protein expression of Nur77 in MH7A cells, while EA promoted Nur77 expression, and transfection of si-Nur77 decreased Nur77 expression (Figures 6(a) and 6(b)). Moreover, EA treatment inhibited proliferation (Figure 6(c)) and promoted apoptosis (Figures 6(d) and 6(e)) in TNF-*α*-treated MH7A cells, while Nur77 knockdown reversed these effects. Furthermore, TNF-*α* treatment increased the levels of IL-6 and IL-1*β* in MH7A cells, while EA inhibited the levels of IL-6 and IL-1*β*, which were reversed by Nur77 knockdown (Figures 6(f) and 6(g)). Additionally, TNF-*α* treatment decreased SOD level (Figure 6(h)) and increased MDA level in MH7A cells (Figure 6(i)), while EA treatment increased SOD level and reduced MDA level, and Nur77 knockdown reversed these effects.

### 3.7. EA Alleviated RA Progression in CIA Rats

**Figure 7:**
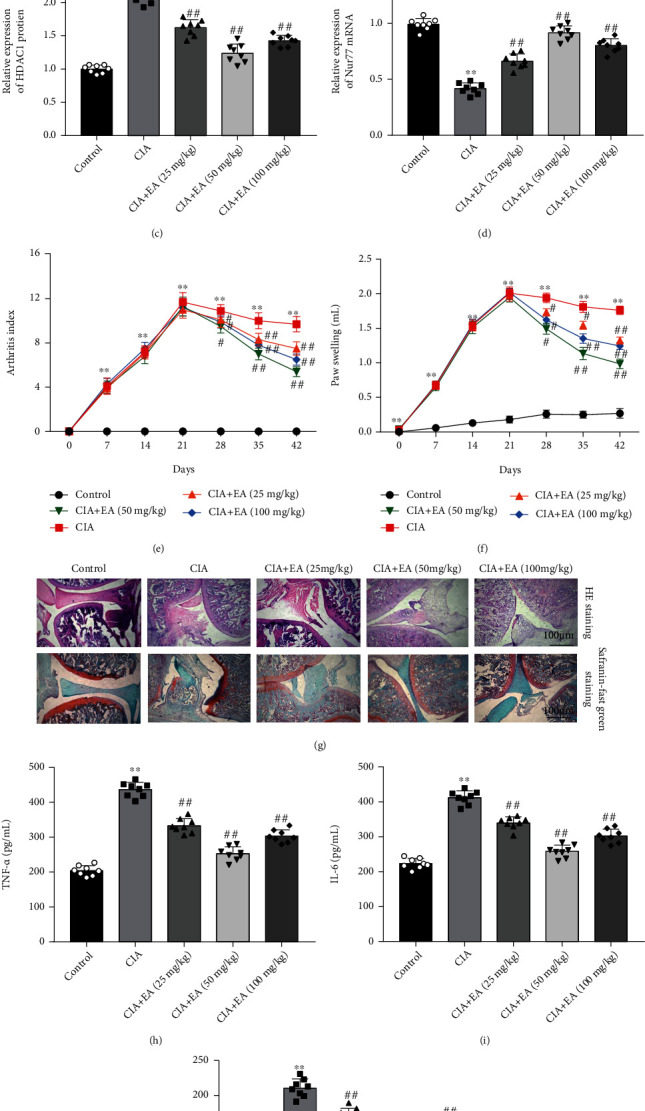
EA alleviated RA progression in CIA rats. Wistar rats were randomly divided into five groups (*n* = 8 per group): control, CIA, CIA + EA (25 mg/kg), CIA + EA (50 mg/kg), and CIA + EA (100 mg/kg). The rats were immunized by intradermally injecting the collagen emulsion to establish CIA rat model, and EA was orally administered. (a–d) The expression of MTA1, HDAC1, and Nur77 in synovial tissues of rats was measured by using Western blot analysis. (e) The arthritis index of rats in each group was detected once a week. (f) The paw swelling of rats in each group was detected once a week. (g) Effects of EA on histopathological changes in the synovial tissues of CIA rats, using HE staining. (h–j) The levels of TNF-*α*, IL-1*β*, and IL-6 in synovial tissues were examined by using ELISA. Data from at least three replicate experiments were presented as mean ± SD. ^∗∗^*P* < 0.01 compared with the control group; ^#^*P* < 0.05 and ^##^*P* < 0.01, compared with the CIA group.

To explore the effect of EA in RA progression *in vivo*, a CIA mouse model was constructed by injecting collagen emulsion, and different concentrations of EA (25, 50, and 100 mg/kg) were given orally. As shown in Figures 7(a)–7(c), we confirmed the increased MTA1 and HDAC1 expression and decreased Nur77 expression in synovial tissues of CIA rats, while this expression pattern was reversed by EA treatment (Figures 7(a)–7(d)). Moreover, compared with the control group, the bilateral paw joints of rats showed obvious redness and swelling, and the arthritis index (Figure 7(e)) and paw swelling degree (Figure 7(f)) were increased continuously and reached the highest level on day 21 after modeling. Compared with the CIA group, both the arthritis score and paw swelling were significantly decreased in EA-treated CIA rats. As shown in Figure 7(g), synovial histopathological evaluation showed that the control group presented a clear and smooth tissues without inflammatory cell infiltration and synovial hyperplasia, and the CIA group exhibited obvious inflammatory cell infiltration and synovial hyperplasia, whereas EA treatment effectively alleviated inflammatory cell infiltration and synovial hyperplasia in CIA rats. Additionally, we found that the levels of inflammatory cytokines TNF-*α*, IL-6, and IL-1*β* were significantly elevated in synovial tissues of CIA rats, whereas EA treatment significantly decreased their levels (Figures 7(h)–7(j)). Importantly, we observed that the protective effect of 50 mg/kg EA is better than that of 25 mg/kg and 100 mg/kg. Taken together, we suggested that EA alleviated RA progression in CIA rats.

## 4. Discussion

At present, various traditional disease-modifying antirheumatic drugs and biological agents are used for the treatment of RA. Unfortunately, many RA patients respond poorly to the general drugs, and there are no effective cures for RA. Emerging evidence indicated that FLSs exhibit abnormal proliferation and insufficient apoptosis, which drives synovial hyperplasia and inflammatory response in the pathophysiology of RA [3, 4]. Current evidence indicated that natural products inhibited the biological behaviors of activated FLSs, which might provide new insights into the treatment of RA [5, 17]. The natural polyphenol EA has been reported to have antioxidant, anti-inflammatory, and anticancer biological activities. EA prevented the development of lung and heart injuries though ameliorating lung inflammation and heart oxidative stress in elastase-induced emphysema model in rats [18]. Interestingly, it was previously reported that EA alleviated adjuvant induced arthritis in the mouse model mice by downregulation of proinflammatory cytokines and upregulation of anti-inflammatory cytokines [7]. Moreover, EA attenuated the severity of adjuvant-induced arthritis in rats through regulating inflammatory signals, oxidative stress, and angiogenesis [8]. Based on the above information, we investigated the underlying roles of EA in RA progression *in vitro* and *in vivo*, and our results showed that EA inhibited proliferation, inflammation, and oxidative stress and promoted apoptosis in FLS MH7A cells. Additionally, EA alleviated the severity of RA in CIA rat model.

MTA1 and HDAC1 proteins are essential components of the NuRD complex that mediates gene transcriptional repression [13]. It was reported that MTA1 was upregulated in human rheumatoid synovial tissues, and MTA1 knockdown reduced the 4-hydroxynonenal-induced mRNA levels of proinflammatory cytokines including IL-1*β*, TNF-*α*, and IL-6 in MH7A cells [12]. HDAC1 is a type of histone deacetylases and plays an inhibitory effect on gene transcription by regulating the acetylation of histone lysine residues and nonhistone proteins. HDAC1 was found to be upregulated in RA synovial fibroblasts, and HDAC1 knockdown resulted in reduced proliferation, invasion, and migration in RA synovial fibroblasts. Moreover, HDAC1 inhibition reduced joint swelling, cartilage, and bone damage in joint tissues of CIA rats [19]. More importantly, nonselective HDAC inhibitors were found to have anti-inflammatory properties in both *in vitro* and *in vivo* models of RA [20, 21]. Similarly, our results demonstrated that MTA1 was upregulated in TNF-*α*-treated MH7A cells, and MTA1 knockdown inhibited proliferation, inflammation, and oxidative stress and promoted apoptosis in MH7A cells. Moreover, we verified the interaction between MTA1 and HDAC1 in MH7A cells, and HDAC1 overexpression reversed the effect of MTA1 knockdown on MH7A cell biological behaviors. Interestingly, current studies provided the evidence that polyphenols played functional roles in human physiological and pathological processes via regulating the MTA1/HDAC1 complex. Pterostilbene suppressed the growth and invasion of hepatocellular carcinoma through inhibiting the MTA1/HDAC1/NuRD complex and promoting PTEN acetylation [14]. Resveratrol downregulated the MTA1/HDAC complex and facilitated PTEN acetylation, thus blocking the PTEN/Akt pathway and inhibiting the progression of prostate cancer [15]. In this study, we revealed that EA downregulated the MTA1/HDAC complex in MH7A cells, and MTA1 overexpression reversed the preventive effects of EA on proliferation, insufficient apoptosis, inflammation, and oxidative stress.

Orphan nuclear receptor Nur77, also known as nuclear receptor subfamily 4 group A member 1 (NR4A1), is a member of the nuclear receptor superfamily, which is a key negative regulator in inflammatory responses [22]. In particular, NR4A1 has been shown to have a protective function in inflammatory joint disease [23]. For instance, NR4A1 reactivation by its agonist cytosporone B could inhibit IL-1*β* induced chondrocyte inflammation, and injection of cytosporone B protected cartilage damage and alleviated osteoarthritis in rat osteoarthritis model [24]. More importantly, current available research elucidated that NR4A receptors modified the activity of FLSs to regulate synovial tissue hyperplasia, pathological angiogenesis, and cartilage turnover [25], which indicated that Nur77 might be involved in the progression of RA. Besides, Nur77 was identified as an acetylated protein, and HDAC1 decreased the acetylation level, transcriptional activity, and protein level of Nur77 [15]. Consistently, we showed that the mRNA and protein expression of Nur77 is regulated by HDAC1-mediated deacetylation in MH7A cells. EA upregulated Nur77 expression by inhibiting MTA1/HDAC1 complex, and Nur77 knockdown reversed the preventive effects of EA on proliferation, insufficient apoptosis, inflammation, and oxidative stress, indicating the protective roles of Nur77 in RA progression.

Taken together, our findings suggested that EA downregulated MTA1/HDAC1 complex and promoted HDAC1 deacetylation-mediated Nur77 expression in MH7A cells, thus inhibiting proliferation, inflammation, and oxidative stress and promoting apoptosis. Further *in vivo* studies showed that EA alleviated the severity of RA in CIA rat model.

## Data Availability

All the data were presented in the article.

## References

[1] Cai P., Jiang T., Li B. (2019). Comparison of rheumatoid arthritis (RA) and osteoarthritis (OA) based on microarray profiles of human joint fibroblast-like synoviocytes. *Cell Biochemistry and Function*.

[2] Doody K. M., Bottini N., Firestein G. S. (2017). Epigenetic alterations in rheumatoid arthritis fibroblast-like synoviocytes. *Epigenomics*.

[3] Bustamante M. F., Garcia-Carbonell R., Whisenant K. D., Guma M. (2017). Fibroblast-like synoviocyte metabolism in the pathogenesis of rheumatoid arthritis. *Arthritis Research & Therapy*.

[4] Han D., Fang Y., Tan X. (2020). The emerging role of fibroblast-like synoviocytes-mediated synovitis in osteoarthritis: an update. *Journal of Cellular and Molecular Medicine*.

[5] Pan D., Li N., Liu Y. (2018). Kaempferol inhibits the migration and invasion of rheumatoid arthritis fibroblast-like synoviocytes by blocking activation of the MAPK pathway. *International Immunopharmacology*.

[6] Liu W., Li J. (2019). Theaflavin-3, 3’-digallate attenuates rheumatoid inflammation in mice through the nuclear factor-*κ*B and MAPK pathways. *Archivum Immunologiae et Therapiae Experimentalis*.

[7] Allam G., Mahdi E. A., Alzahrani A. M., Abuelsaad A. S. (2016). Ellagic acid alleviates adjuvant induced arthritis by modulation of pro- and anti-inflammatory cytokines. *Central European Journal of Immunology*.

[8] Fikry E. M., Gad A. M., Eid A. H., Arab H. H. (2019). Caffeic acid and ellagic acid ameliorate adjuvant-induced arthritis in rats via targeting inflammatory signals, chitinase-3-like protein-1 and angiogenesis. *Biomedicine & Pharmacotherapy*.

[9] Song Q., Wang B., Liu M. (2019). MTA1 promotes the invasion and migration of oral squamous carcinoma by inducing epithelial-mesenchymal transition via the hedgehog signaling pathway. *Experimental Cell Research*.

[10] Li Y. H., Zhong M., Zang H. L., Tian X. F. (2020). MTA1 promotes hepatocellular carcinoma progression by downregulation of DNA-PK-mediated H1.2T146 phosphorylation. *Oncology*.

[11] Sen N., Gui B., Kumar R. (2014). Physiological functions of MTA family of proteins. *Cancer and Metastasis Reviews*.

[12] Wang H., Dong B. W., Zheng Z. H., Wu Z. B., Li W., Ding J. (2016). Metastasis-associated protein 1 (MTA1) signaling in rheumatoid synovium: regulation of inflammatory response and cytokine-mediated production of prostaglandin E_2_ (PGE_2_). *Biochemical and Biophysical Research Communications*.

[13] Link S., Spitzer R. M. M., Sana M. (2018). PWWP2A binds distinct chromatin moieties and interacts with an MTA1-specific core NuRD complex. *Nature Communications*.

[14] Qian Y. Y., Liu Z. S., Yan H. J., Yuan Y. F., Levenson A. S., Li K. (2018). Pterostilbene inhibits MTA1/HDAC1 complex leading to PTEN acetylation in hepatocellular carcinoma. *Biomedicine & Pharmacotherapy*.

[15] Dhar S., Kumar A., Li K., Tzivion G., Levenson A. S. (2015). Resveratrol regulates PTEN/Akt pathway through inhibition of MTA1/HDAC unit of the NuRD complex in prostate cancer. *Biochimica et Biophysica Acta*.

[16] Wang C., Liu G., Dou G. (2021). Z-Ligustilide selectively targets AML by restoring nuclear receptors Nur77 and NOR-1-mediated apoptosis and differentiation. *Phytomedicine*.

[17] Lu J., Yang J., Zheng Y., Fang S., Chen X. (2019). Resveratrol reduces store-operated Ca2+ entry and enhances the apoptosis of fibroblast-like synoviocytes in adjuvant arthritis rats model via targeting ORAI1-STIM1 complex. *Biological Research*.

[18] Mansouri Z., Dianat M., Radan M., Badavi M. (2020). Ellagic acid ameliorates lung inflammation and heart oxidative stress in elastase-induced emphysema model in rat. *Inflammation*.

[19] Hawtree S., Muthana M., Wilkinson J. M., Akil M., Wilson A. G. (2015). Histone deacetylase 1 regulates tissue destruction in rheumatoid arthritis. *Human Molecular Genetics*.

[20] Zhang Y., Zhang B. (2016). Trichostatin a, an inhibitor of histone deacetylase, inhibits the viability and invasiveness of hypoxic rheumatoid arthritis fibroblast-like synoviocytes via PI3K/Akt signaling. *Journal of Biochemical and Molecular Toxicology*.

[21] Choudhary N., Gupta R., Bhatt L. K. (2020). Anti-rheumatic activity of Phenethyl isothiocyanate via inhibition of histone deacetylase-1. *Chemico-Biological Interactions*.

[22] Rodríguez-Calvo R., Tajes M., Vázquez-Carrera M. (2017). The NR4A subfamily of nuclear receptors: potential new therapeutic targets for the treatment of inflammatory diseases. *Expert Opinion on Therapeutic Targets*.

[23] Hamers A. A. J., Argmann C., Moerland P. D. (2016). Nur77-deficiency in bone marrow-derived macrophages modulates inflammatory responses, extracellular matrix homeostasis, phagocytosis and tolerance. *BMC Genomics*.

[24] Xiong Y., Ran J., Xu L. (2020). Reactivation of NR4A1 restrains chondrocyte inflammation and ameliorates osteoarthritis in rats. *Frontiers in Cell and Developmental Biology*.

[25] Crean D., Murphy E. P. (2021). Targeting NR4A nuclear receptors to control stromal cell inflammation, metabolism, angiogenesis, and tumorigenesis. *Frontiers in Cell and Developmental Biology*.

